# The Effect of Sodium Glucose Cotransporter 2 Inhibitors From a Human Genetic Perspective

**DOI:** 10.3389/fgene.2021.658012

**Published:** 2021-03-19

**Authors:** Xing-zi Liu, Hong Zhang

**Affiliations:** ^1^Renal Division, Department of Medicine, Peking University First Hospital, Beijing, China; ^2^Key Laboratory of Renal Disease, Ministry of Health of China, Beijing, China; ^3^Key Laboratory of Chronic Kidney Disease Prevention and Treatment (Peking University), Ministry of Education of China, Beijing, China

**Keywords:** SGLT2 inhibitor, genetic, omics, eQTL, drug targets

## Introduction

The increasing prevalence of type 2 diabetes mellitus (T2DM) is the most common cause of chronic kidney disease (CKD) during the recent decades (Ogurtsova et al., 2017). Since 2001, only renin-angiotensin system blockers were approved treatment for renoprotection in patients with T2DM (Brenner et al., 2001). Sodium glucose cotransporter 2 (SGLT2) inhibitors were designed as glucose-lowering agents for the treatment of T2DM. Intriguingly, SGLT2 inhibitors were recently found to beyond glucose lowering that included potential renoprotective and cardiovascular benefits in patients with and without T2DM (Perkovic et al., 2019; Heerspink et al., 2020).

Current development of large-scale omics offers an amount of publicly available omics databases, which provided the transcriptome and proteomes spatial characterization in the different tissues of the human body and expression quantitative trait loci (eQTLs) in the kidney compartments (Gillies et al., 2018; Qiu et al., 2018). Previously, we integrated the omics databases of human angiotensin converting enzyme II, which was identified as the target functional receptor of SARS-CoV-2, and predicted the potential universal kidney susceptibility to SARS-CoV-2 in general population (Zhang and Zhang, 2020; Zhang et al., 2020). Perhaps, the genetic strategy used to explore the roadmap for kidney involvement of SARS-CoV-2 infection, including genetic analysis of the spatial distribution of human gene expression and its genetic determinants in kidney, offers a promising opportunity to predict the success of drug targets. In this study, we validated the reliability and availability of the genetic strategy by taking SGLT2 inhibitors as an example by comparing with the published randomized controlled trials.

## Putative Less Adverse Effect of SGLT2 Inhibitors

**Figure 1:**
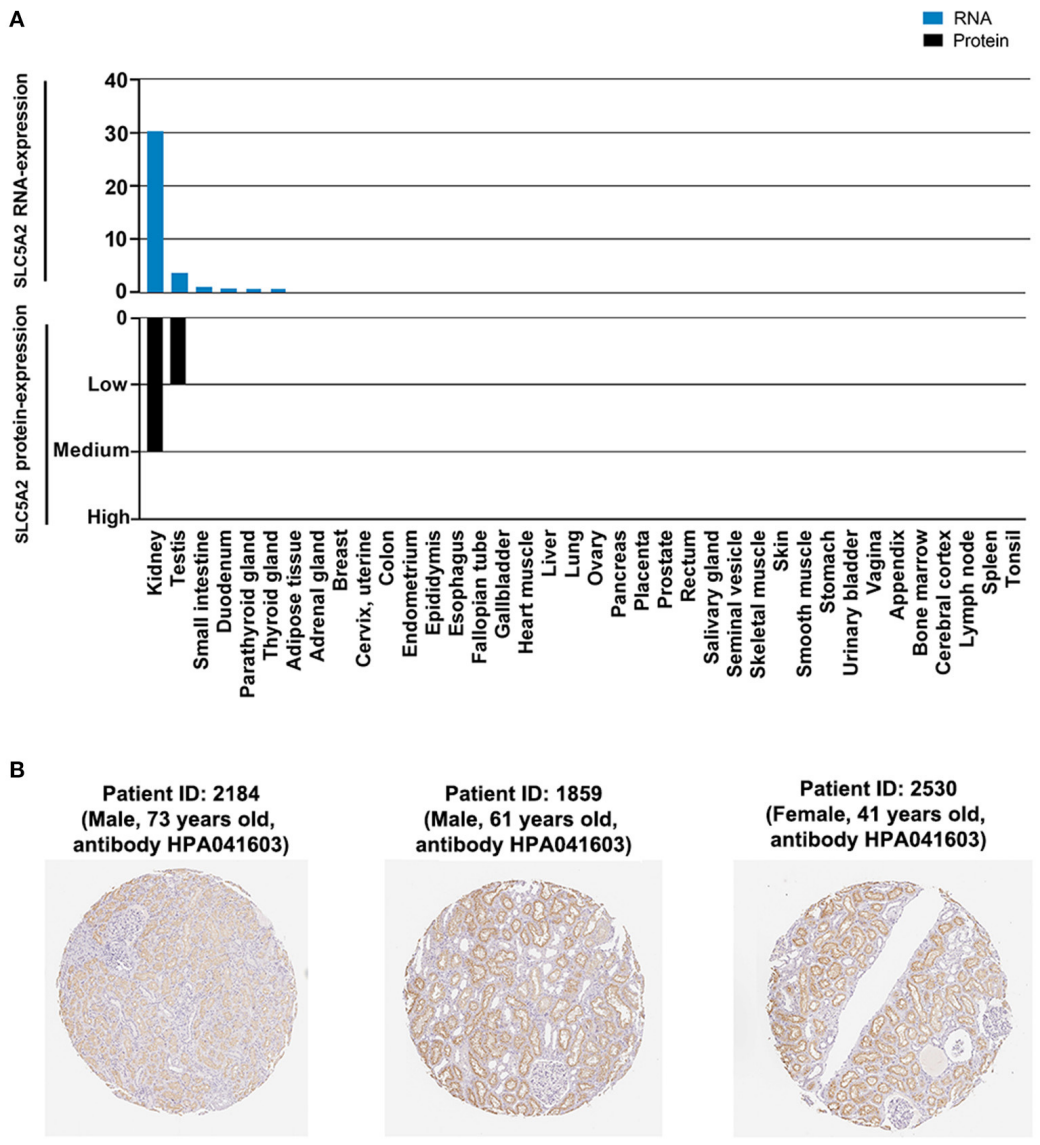
Human SLC5A2 was highly and specifically expressed in kidney tissue, especially tubules but not in glomeruli. **(A)** The RNA- and protein-level distribution of SLC5A2 in human tissues. **(B)** Protein expression of SLC5A2 in normal kidney tissue from three patients. These results were derived from the Human Protein Atlas database (https://www.proteinatlas.org/).

SGLT2 gene (Aliase: SLC5A2) was reported to be the drug target for SGLT2 inhibitors. Thus, the spatial distribution of human SLC5A2 would provide insights into the target tissues of SGLT2 inhibitors, which in turn help predict the potential of target effect. To do so, we searched the gene and protein expression levels and tissue-based spatial characterization of human SLC5A2 in the Human Protein Atlas database (https://www.proteinatlas.org/) (Uhlen et al., 2015), which provided transcriptomic and antibody-based proteomic information of more than 90% of the putative protein-coding genes in 32 different tissues and organs in general populations. The result showed that human SLC5A2 was highly and specifically expressed in kidney (Figure 1A). Moreover, in kidney, human SLC5A2 was specifically highly expressed in tubules but not in glomeruli (Figure 1B). The highly selective expression of SLC5A2 in kidney tubules was in line with the relatively less adverse effects of SGLT2 inhibitors comparted with placebo (Perkovic et al., 2019).

## Clinical Efficacy of SGLT2 Inhibitors in Non-Diabetic Settings

Although diabetic nephropathy is the most common cause of CKD worldwide, our Mendelian randomization study of T2DM and glucose-related phenotypes on CKD suggested the glucose-independent effect of T2DM on CKD (Zheng et al., 2020). In line with our genetic findings, the CREDENCE trial (Canagliflozin and Renal Events in Diabetes with Established Nephropathy Clinical Evaluation) showed a lower risk of kidney failure and cardiovascular events among T2DM patients receiving Canagliflozin than among those receiving placebo (Perkovic et al., 2019), while overall mean change of hemoglobin A1c (HbA1c) in Canagliflozin group compared with placebo was only −0.25%. Follow-up analysis splitting by baseline HbA1c categories, Cannon et al. (2020) reported the similar renal and cardiovascular protective effects of Canagliflozin. The modest glucose lowering effect and similar effects across HbA1c categories suggested the glucose-independent mechanism of renal and cardiovascular benefits of SGLT2 inhibitors. Indeed, a recent landmark DAPA-CKD trial (The Dapagliflozin and Prevention of Adverse Outcomes in Chronic Kidney Disease) (Heerspink et al., 2020) reported the success of SGTL2 inhibitors in non-diabetic settings.

## Prediction of Efficacy of SGLT2 Inhibitors Across Populations

We first reviewed the studies to identify the functional variants at SLC5A2 gene. Some functional variants at SLC5A2 gene were found to be associated with familial renal glucosuria (Yu et al., 2020) and the risk of glucosuria (Benonisdottir et al., 2019). However, most of these functional variants were private and rare across populations (highest minor allele frequency < 1%), which can be observed in any populations in 1000 Genomes Project Consortium et al. (2015). To identified the genetic determinants of human SLC5A2 gene expression in kidney tubulointerstitial, we then further searched the NephQTL database (http://nephqtl.org/) (Gillies et al., 2018), which contained thousands of kidney specific eQTLs of micro dissected tubulointerstitial compartments (*n* = 166) from patients with proteinuric kidney diseases, such as minimal change disease, focal segmental glomerular sclerosis, and membranous nephropathy, and the Human Kidney eQTL Atlas database (http://susztaklab.com/eqtl) (Qiu et al., 2018), which contained eQTLs of micro dissected tubulointerstitial compartments (*n* = 119) from kidney of healthy human of undergoing surgical nephrectomy. In NephQTL database, although 154 variants with *P* < 0.05 were found with the smallest *P*-value of 8.73 × 10^−3^, none of the variants achieved the Bonferroni-corrected threshold of *P* < 3.25 × 10^−4^. In consistent with this, the Human Kidney eQTL Atlas database showed no significant eQTL of SLC5A2 expression in tubulointerstitial compartments, suggesting that the genetic variants are less likely to affect the gene expression of human SLC5A2 in kidney tubules. These genetic evidences might support the potential wide benefits of SGLT2 inhibitors across different populations. Notably, patients in the CREDENCE and DAPA-CKD trials were recruited at more than 400 sites in over 20 countries, the wide efficacy and safety of SGLT2 inhibitors among the different populations in the trials validated our prediction from the genetic perspective.

## Discussion

Previous phenome-wide association study in combination with tissue-specific gene expression and eQTL showed that the presence of eQTL in multiple tissues resulted in more unique phenotypes driven by genome-wide association loci and drugs delivered to multiple tissues can induce several side effects (Duffy et al., 2020). More importantly, recent establishment of an amount of publicly available databases based large-scale omics provided a timely opportunity to understand the effects and safety of target drugs across populations. The SGLT2 inhibitors were the breakthrough in renal and cardiovascular benefits. Taking SGLT2 inhibitors as an example, in the one hand, the spatial characteristics of RNA and protein expression of human SLC5A2 combining with kidney specific eQTL analysis indicated that SLC5A2 gene specifically expressed and detected in the kidney tubules, and no clear genetic determinants were reported, suggesting the potential wide renal effects and safety of SGLT2 inhibitors in patients across different ethnic populations. In the other hand, SLC5A2 was found to not express in cardiovascular tissue, but previous study reported that SGLT2 inhibitors could indirectly reduce cardiac preload through promoting osmotic diuresis, resulting in reduction of volume overload and improving cardiovascular function (Cherney et al., 2014). Therefore, the potential cardiovascular protective effects of SGLT2 inhibitors might be wide across different populations. Overall, our genetic strategy provided a possible genetic pipeline to help evaluate the success of potential drug targets.

## Author Contributions

HZ conceived the study. X-zL drafted the manuscript. All authors contributed to the article and approved the submitted version.

## Conflict of Interest

The authors declare that the research was conducted in the absence of any commercial or financial relationships that could be construed as a potential conflict of interest.
